# Analysis of an Outbreak of Hemorrhagic Fever with Renal Syndrome in College Students in Xi’an, China

**DOI:** 10.3390/v6020507

**Published:** 2014-01-29

**Authors:** Chaofeng Ma, Zengguo Wang, Shen Li, Yuan Xing, Rui Wu, Jing Wei, Muhammad Nawaz, Huaiyu Tian, Bing Xu, Jingjun Wang, Pengbo Yu

**Affiliations:** 1 Xi’an Centers for Disease Control and Prevention, Xi’an 710054, China; E-Mail: mark7447@tom.com (C.M.); william_wzg@126.com (Z.W.); tjxxyy@163.com (Y.X.); wur2003@163.com (R.W.); 2 Shaanxi Centers for Disease Control and Prevention, Xi’an 710054, China, E-Mail: lishen12345@163.com (S.L.); weijingnwu@gmail.com (J.W.), jingjunwang@china.com (J.W.); 3 Department of Microbiology, Faculty of Veterinary Sciences, University of Veterinary and Animal Sciences, Lahore 54600, Pakistan; E-Mail: muhammad.nawaz@uvas.edu.pk; 4 College of Global Change and Earth System Science, Beijing Normal University, Beijing 100875, China; E-Mail:tianhuaiyu@gmail.com (H.T.); bingxu@tsinghua.edu.cn (B.X.)

**Keywords:** Hantaan virus, hemorrhagic fever with renal syndrome, outbreak, Xi’an City

## Abstract

The aim of the present study was to analyze an outbreak of hemorrhagic fever with renal syndrome (HFRS), caused by a Hantavirus, in college students in the northern urban area of Xi’an in 2012. The outbreak affected six students and included two deaths. The epidemiological survey revealed that both of the deceased cases were misdiagnosed initially, and treatment was delayed. Furthermore, a higher rodent population density and lower HFRS vaccine coverage were observed in the affected area, which indicates a possible role in the outbreak. Rattus norvegicus (Rn) and Mus musculus (Mm) were the predominant host populations in the area. Genotyping revealed that all HVs from patients and rodents were Hantaan virus (HTNV). Sequence analysis of the S segments revealed that the HTNVs reported in this study had high similarity with strains reported in 2011 and 1985, but these viruses diverged from a strain isolated in 1984 and the HTNV prototype strain 76-118. Detection of anti-HV IgG and amplification of the S segment of HTNV from a non-natural HTNV reservoir indicates that further investigations by increased rodent trapping are necessary.

## 1. Introduction

Hemorrhagic fever with renal syndrome (HFRS) is caused by hantaviruses in the Asia-Pacific area. The clinical manifestations of HFRS are fever, hemorrhage and varying degrees of renal and hepatic dysfunction [1]. The HVs that cause HFRS include: Hantaan virus (HTNV), Seoulvirus (SEOV), Puumalavirus (PUUV) and Dobrava virus (DOBV) [2,3]. The main natural reservoir of hantaviruses are murid rodents (order Rodentia; family Muridae; subfamilies Murinae, Arvicolinae and Sigmodontinae). The host rodents, which have no disease manifestations, share a long period of co-evolution with hantaviruses. People are usually infected by inhalation of aerosolized rodent excreta, such as saliva, urine or feces, or from a rodent bite or blood transfusion [4].

Shaanxi province, which is located in northwestern China, is one of the most seriously affected areas since the first case of HFRS was reported in 1955. Shaanxi province has the highest number of HFRS cases in China since 2010. Recently, an HFRS outbreak occurred in a college in Xi’an, the capital of Shaanxi Province, in which six students were infected and two died.

On Nov. 13th, 2012, three HFRS cases (two lab confirmed and one suspicious) from a college (college A) located in northern Xi’an were reported to the Xi’an Center for Disease Control and Prevention (CDC). From the same college, three more cases were observed on Nov. 17th, Nov. 21st and Dec. 2nd. All six patients (three males and three females) were students from the same college with an average age of 21.3 years (19–23). The main symptoms of the patients were fever (highest 39.2–40.7 °C), backache, hemorrhage and acute tubulointerstitial nephritis. The deceased cases both developed into shock, necrotizing glomerulonephritis and multi-organ failure.

The first patient developed fever on Nov. 4th. She went to a private hospital three days later, where she was diagnosed and treated for an upper respiratory tract infection. However, when her condition did not improve, the patient was subsequently admitted to the Department of Infectious Diseases of a professional hospital on Nov. 10th. In the professional hospital, the patient was diagnosed and treated for the oliguric phase of HFRS. She died after seven days of hospitalization. The disease course of the second patient was similar to the course of the first. This patient had onset of fever on Nov. 5th and self-treated for flu for three days. The self-treatment was followed by treatment for an upper respiratory tract infection in the same private hospital on Nov. 8th. He was then admitted to the professional hospital on Nov. 12th and finally died on Nov. 24th. The third patient developed fever on Nov. 11th and went directly to the professional hospital on the following day. Here, she was diagnosed as a possible HFRS case. Because the first three cases were from the same college (A), the hospital reported the situation to the Xi’an CDC. After the first death, each student of college A that developed fever was examined for routine blood and urine chemistry and screened for anti-HV IgM. Possible HFRS cases were sent to the professional hospital. In the following days, three more HFRS patients were found in college A.

## 2. Experimental

### 2.1. Rodent Population Density Survey and Sample Collection

**Figure 1:**
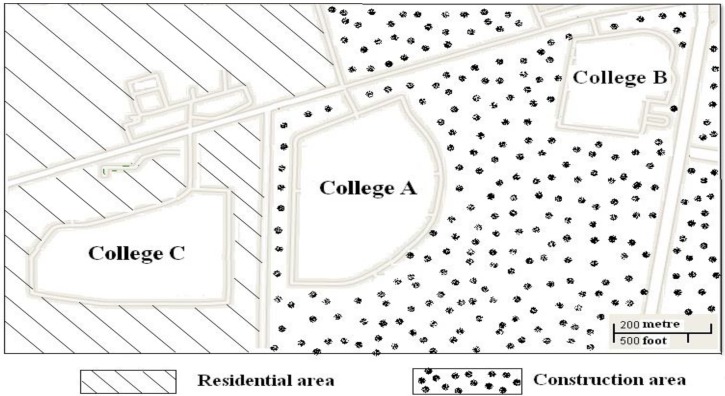
Map of locations of colleges.

College A is located in the northern part of Xi’an municipal close to two other colleges (B and C) (Figure 1). To determine the size of the rodent population, 500 mouse traps (baited with peanuts) were placed 5 m apart in each of the colleges. Trapped rodents were identified according to previously described criteria and transported to the laboratory in the Xi’an CDC [5]. Lung tissues from the rodents were obtained and stored immediately at −80 °C until further processing. Sera of the rodents were collected and stored at −20 °C for antibody detection. Sera of the six patients were also collected from the hospital to confirm HFRS. The sera of 177 apparently healthy students, who had close contact with these six patients, were also collected for HV Ab detection.

### 2.2. Detection of Antibody against HV in the Sera

For all sera from the six patients and 177 healthy students, anti-HV IgM and IgG were analyzed using the Hantavirus IgG/IgM Combo Test (Colloidal gold immunochromatographic assay, Xiamen Biotech, China) according to the manufacturer’s instructions. The Hantavirus IgG/IgM Combo Test can detect anti-HV IgM and IgG simultaneously. The sera from the healthy students and the rodents were also tested for anti-HV IgG using the Hantavirus IgG ELISA Kit (Beijing Wantai, Beijing, China). Briefly, the test was performed by adding diluted serum (1:100 in phosphate-buffered saline) to an HV antigen (nucleocapsid protein) coated plate. Following the incubation, captured IgG was detected using Goat Anti-Human/Anti-Mouse IgG (H+L) antibody labeled with horseradish peroxidase (Promega, Beijing, China). The test results were determined with a cutoff value (0.3).

### 2.3. Reverse Transcriptase-PCR for the Detection of HV RNA

From the sera of patients, viral RNAs were extracted using the QIAamp viral RNA mini kit according to the manufacturer’s instructions (Qiagen, Hilden, Germany). Lung tissues of the rodents were homogenized with a Tissue Lyser (Qiagen, Hilton, Germany), and total RNAs were extracted with Trizol reagent according to the manufacturer’s instructions (Invitrogen, Beijing, China). The cDNAs were synthesized by Revert Aid TM First Strand cDNA Synthesis kit using random hexamers according to the manufacturer’s instructions (Fermentas, Shenzhen, China). For HV genotyping, the medium (M) segment of the genome was amplified by nested PCR using the HV-G-F and HV-G-R primer pair for the initial round of PCR and the HTNV-M-F, HTNV-M-R and SEO-M-F, SEOV-M-R primers for the second round of amplification [6].

### 2.4. Amplification and Sequencing of the S Segments and Phylogenetic Analysis

The cDNAs of samples that were strongly positive by genotyping PCR were used to amplify the three overlapping parts of the entire S segment (primers are available upon request). The products were sequenced using the Big Dye Terminator v 3.1 Cycle Sequencing Kit on ABI-PRISM3730 (Applied Biosystems, Carlsbad, CA, USA) by Sangon Biotech Co., Ltd (Shanghai, China). The nucleotide sequences and deduced amino acid sequences were analyzed using the BioEdit Program [7]. A phylogenetic tree was constructed using the neighbor-joining and maximum likelihood methods with 1,000 bootstrap replicates [8]. Nucleotide and amino acid similarities were calculated by the DNA Star program (DNASTAR, Madison, WI, USA). Other HV sequences used to construct the phylogenetic tree were retrieved from GenBank [9].

### 2.5. Statistical Analysis

Statistical analysis was performed using SPSS 13.0 (SPSS, Chicago, IL, USA). Categorical variables were compared by chi-square. A *p* value of <0.05 was considered to be statistically significant. All statistical operations were 2-tailed.

## 3. Results

### 3.1. Clinical Manifestations

The clinical signs and symptoms of HFRS for the patients in this outbreak are summarized in Table 1. All six individuals had the typical manifestations of HFRS. Additionally, the deceased patients developed necrotizing glomerulonephritis and multiple organ failure. For the treatment of HFRS, ribavirin was administered by intravenous drip (15 mg/kg body weight, twice a day) for two, four, four, three and five days for cases number 2–5, respectively. The first case did not receive the ribavirin treatment because of the delayed diagnosis and worsened condition. Treatment with ribavirin was started immediately after the diagnosis of HFRS and stopped once the body temperature of the case was normalized. The last four patients (3–6) fully recovered in three weeks.

**Table 1:** Clinical symptoms and signs of HFRS for patients in this outbreak.

Clinical Data	Patient No.					
	1 *	2 *	3	4	5	6
Sex	F	M	F	F	M	M
Age(Year)	23.3	19.6	22.4	19.1	20.6	22.8
Signs and symptoms						
Fever (days)	7	9	4	4	3	5
Peak temperature (°C)	39.4	39.8	39.6	40.2	39.2	40.7
Chills	Y	Y	N	N	N	N
Malaise	Y	Y	Y	Y	Y	Y
Fatigue	Y	Y	Y	Y	Y	Y
Myalgia	Y	N	N	N	N	N
Abdominal pain	Y	Y	N	N	N	N
Nausea	Y	Y	Y	Y	Y	Y
Vomiting	Y	Y	N	N	N	N
Backache	Y	Y	Y	Y	Y	Y
Chest pain	Y	Y	Y	Y	Y	Y
Eyeball pain	Y	Y	Y	Y	Y	Y
Diffuse reddening	Y	Y	Y	Y	Y	Y
Bradycardia	N	N	Y	N	Y	N
Photophobia	N	N	Y	N	N	N
Pharynx enanthema	N	N	N	N	N	Y
Petechia	Y	Y	Y	Y	Y	Y
Conjunctival hemorrhages	Y	Y	Y	Y	Y	Y
Thrombocytopenia	Y	Y	Y	Y	Y	Y
Acute tubulointerstitial nephritis	Y	Y	Y	Y	Y	Y
Proteinuria	Y	Y	Y	Y	Y	Y
Shock	Y	Y	N	N	N	N
Hypotension	Y	Y	N	N	N	N
Necrotizing glomerulonephritis	Y	Y	N	N	N	N
Multi-organ hypoperfusion	Y	Y	N	N	N	N
Multi-organ dysfunction	Y	Y	N	N	N	N
Multi-organ failure	Y	Y	N	N	N	N

*: The deceased cases. Note: M, male; F, female; Y, yes; N, no.

### 3.2. Rodent Population Density Survey

A total of 36 rodents including *Rattus norvegicus*, Rn (n = 21), *Mus musculu*s, Mm (n = 14) and *Apodemus agrarius*, Aa (n = 1), were captured using mouse traps (Table 2). The sizes of the rodent populations were 3.6%, 2.8% and 0.8% in Colleges A, B and C, respectively. The rodent population density was significantly lower in College C compared to A and B (χ2 = 9.11 and 5.66, *p* < 0.01 and 0.05).

**Table 2:** Survey of the population density of rodents and IgG antibody against HV.

Campus	Rn		Mm		Aa	
	number	IgG Positive	number	IgG Positive	number	IgG Positive
College A	10	2	7	2	1	0
College B	8	2	6	1	0	0
College C	3	0	1	0	0	0
Total	21	4	14	3	1	0

### 3.3. Detection of Anti-HV Antibody

All six patients were anti-HV IgM positive, while four were anti-HV IgG positive by the Combo Test (Table 3). The sera of all healthy students (n = 177) were negative for both anti-HV IgG and anti-HV IgM according to the Combo Test, while sera of seven healthy students were positive for anti-HV IgG by ELISA. Out of 36 rodent sera, seven sera (four Rn, three Mm) were IgG antibody positive (Table 2).

**Table 3:** Anti-HV IgM and IgG detection and genotyping of HV RNA.

Patients	IgM (Positive days after onset)	IgG	HV RNA genotyping (Positive days after onset)	S segment
1 *	+(6)	-	-	/
2 *	+(7)	+	-	/
3	+(5)	-	HTNV(0)	+
4	+(4)	+	HTNV (1)	/
5	+(4)	+	-	/
6	+(5)	+	HTNV (1)	+

*: The deceased cases.

### 3.4. HV RNA Detection and Sequencing of S Segments from Samples

HV RNA was detected in the sera of three patients and one rodent lung tissue (Rn from college A). Three complete S segments of HVs (two from patients and one from a rodent) were successfully amplified and sequenced. GenBank accession numbers of the sequences reported in this study are KC844226-KC844228.

### 3.5. Phylogenetic Analysis

**Figure 2:**
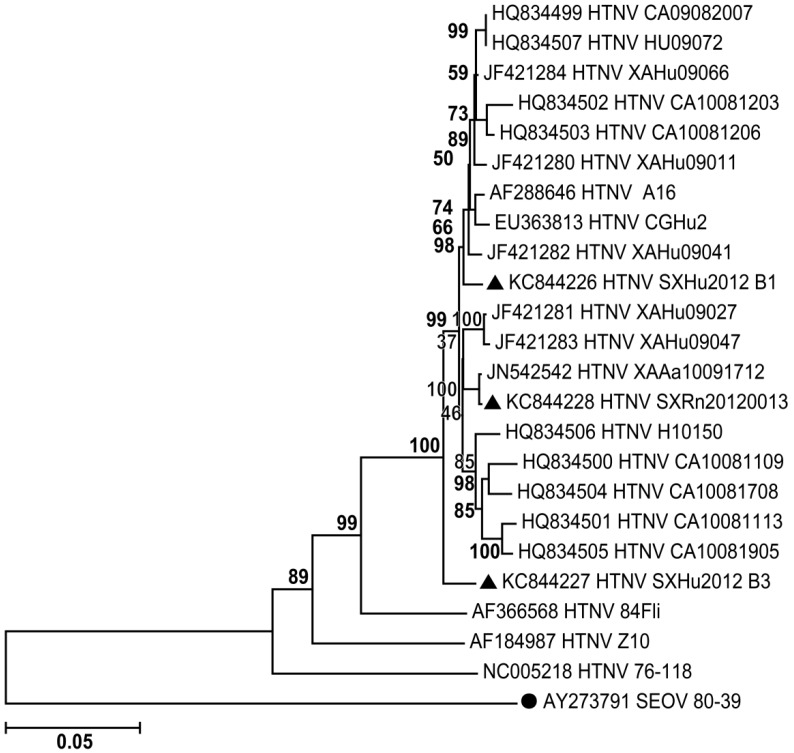
Phylogenetic tree of S segments from related hantaviruses. Mega 5 was used to construct the phylogenetic trees by using the neighbor-joining (NJ) and the maximum likelihood (ML) methods with 1,000 bootstrap replicates. S segment of Seoulvirus (SEOV) was used as an out-group. (●AY273791 SEOV 80–39). ▲The sequences reported in this study.

To establish molecular epidemiological links between the HVs in rodents and HFRS patients, the analysis of the three complete S segments was performed (Figure 2). The S segments that were sequenced in this study were highly similar (97.7%–98.5%). The amino acid sequences of all three S segments were identical. The S segments had high similarity to HTNV (82.7%–98.9%) and lower similarity to SEOV (71.3%–73.1%). Moreover, the phylogenetic analysis of S segments revealed that the sequences reported in this study have high similarity with sequences reported in 2009–2010 (95.8%–99.6%), HTNV/A16 (97.1%–98.4%) and CGHu2 (96.4%–97.6%). The sequences diverged from HTNV/84FLi (91.2%–91.4%), a vaccination strain Z10 (89.2%–89.8%) and the HTNV prototype 76–118 (86.6%–86.7%).

## 4. Discussion

Since the first outbreak in 1955, many outbreaks of HFRS have been reported in China with varying mortality rates (1%–14.2%). In the last 15 years, the death rate has decreased only 1% [10]. Prompt therapeutic interventions, antiviral therapy with ribavirin and bed rest are critical for the treatment of HFRS. Misdiagnosis followed by self or delayed treatment may have contributed to the high mortality rate (33.33%) reported in the present study. After the diagnosis of HFRS, the last four patients were treated with intravenous drips of ribavirin (15 mg/kg body weight, twice a day) until their temperature was normalized, and they recovered in three weeks. Ribavirin is often used for the treatment of hantaviruses in the People’s Republic of China. Clinical trials have shown that ribavirin can significantly reduce the mortality rate of HFRS [11].

HV infection occurs via virus-containing, aerosolized rodent excretions such as urine, feces, or saliva, and people living or working in close contact with infected rodents are at increased risk of infection [12]. The rodent populations sizes were larger in Colleges A and B (construction area) than in College C (residential area). Because Colleges A and B were the only residential sites providing a possible food supply for the rodents, infrastructure development may have led to the perturbation of the rodent habitat and increased the rodent population density. Recently, HFRS outbreaks have also been observed among workers at construction sites in Xi’an (data not published). Rodent habitat perturbation may make the rodents more active and increase the chances of contact with people working or living in the area. A lower rodent population density and no HFRS cases in College C support this hypothesis.

Vaccination and extermination of rodents are the major countermeasures used to control HFRS [6]. Since 2004, the government has provided free HFRS vaccinations for the high-risk population (16–60 years old peasants) in counties that are highly endemic for HFRS. Unfortunately, the college students were not included in the high-risk population of this area. After the outbreak, a survey revealed that the HFRS vaccine coverage rate in students in college B (72%) was much higher compared with College A (0.29%). In 2009, there were some HFRS cases at another branch of college B; since this time, the policy of college B was to vaccinate each of its freshmen for HFRS. No cases of HFRS were reported during that period. The results indicate that the government should enlarge the vaccination programs covering students and building workers to prevent HFRS outbreaks. The rodent population density survey of the area showed that the predominant population was Rn (58.33%) followed by Mm (38.89%) and Aa (2.78%).

Only one Aa (HV negative) was trapped, and HTNV RNA and anti-HV IgG were also detected from Rn and Mm in this survey. Therefore, it is difficult to claim that HTNV carried by Rn or other rodents caused this outbreak because rodent trapping was performed only once. More extensive rodent trapping and further investigations are required to clarify the cause of HFRS in Xi’an.

## 5. Conclusions

This study reports an HFRS outbreak, which was caused by HTNV that affected six students and resulted in two deaths. The high rodent population density and low HFRS vaccine coverage likely contributed to this outbreak. The presence of HTNV in a non-natural host requires further investigation through increased rodent trapping. The expansion of vaccine immunization, early diagnosis and effective treatment may reduce morbidity and mortality of HFRS in the endemic area.
